# siRNA Delivery for Control of Cyclin D1 and E2F1 Expression in Crohn’s Disease

**Published:** 2018-03-31

**Authors:** Ilaria Russo, Albino Carrizzo, Sabrina Bochicchio, Ornella Piazza, Gaetano Lamberti, Anna Angela Barba, Carmine Vecchione, Pio Zeppa, Paola Iovino, Cristina Bucci, Antonella Santonicola, Carolina Ciacci

**Affiliations:** 1 Department of Medicine and Surgery, Scuola Medica Salernitana, University of Salerno, 84081, Baronissi (SA), Italy, IRCCS Neuromed, Pozzilli (IS), 86077, Italy; 2 Department of Industrial Engineering, University of Salerno, via Giovanni Paolo II, 132, Fisciano (SA) – ITALY; 3 Department of Pharmacy, University of Salerno, via Giovanni Paolo II, 132, Fisciano (SA) - ITALY

**Keywords:** IBD (inflammatory bowel disease), Crohn’s disease, Cyclin D1, E2F1, EC-LPS (Lipopolysaccharide from Escherichia Coli), siRNA, nanoliposomes

## Abstract

Evidence in inflammatory bowel diseases (IBD) supports a connection between inflammation and cancer due to the alteration of the cell cycle with loss of control at the G1/S checkpoint. In this study, we analyze the expression and modulation of CyD1 and E2F1 in colon explants from Crohn’s disease (CD) patients.

We used ex vivo culture of colon explants from 4 CD patients and 2 healthy controls, stimulated with lipopolysaccharide from Escherichia Coli (EC-LPS). Commercial siRNAs for CyD1 and E2F1 inhibition were encapsulated in Invivofectamine® and in purposely produced nanoliposomal vectors to silencing CyD1 and E2F1 expression. Western blot analysis was used to investigate the effect of siRNA on CyD1, E2F1 and cyclooxygenase 2 (COX-2) expression. In CD patients colon explants, CyD1 and E2F1 increased after the inflammatory stimulus but siRNA silencing attenuated their expression and controlled the COX-2 expression too. These data represent a prelimiary exploration of in vitro siRNA use.

## INTRODUCTION

The etiopathogenesis of Crohn’s disease (CD) and ulcerative colitis (UC), the major forms of inflammatory bowel diseases (IBD), is still unknown. Many evidence indicates that the IBD-associated tissue damage is due to an exaggerated mucosal inflammatory response [1, 2]. IBD patients show systemic inflammation and some related morbidities [3–7]. mong morbidities, younger patients with CD have also an increased risk of ischemic stroke [8]. Moreover, there is evidence that the chronic inflammation is pro-carcinogenic [9]. In fact, people affected by chronic intestinal inflammation, either with colonic CD or UC, have as well as in other chronic inflammatory conditions, an increased colonic cancer risk [10–12]. However, little is known about the key steps regulating the carcinogenetic process in IBD. Genetic mutations, microsatellite instability, and DNA hypermethylation promote uncontrolled proliferation that leads to crypt elongation and, eventually, adenoma formation and tumor progression [13, 14]. Many studies suggest a relationship between inflammation, cell cycle progression and cancer.

In the relation between cancer and inflammation, NF-κB plays an important role by orchestrating inflammatory responses, cell survival and growth [11]. Experimental data evidence the involvement of NF-κB in colorectal carcinogenesis. NF-κB potentiates tumor proliferation and growth by upregulating the phosphoinositide 3-kinase (PI3K)/Akt cascade and crucial cell-cycle (cyclin-D1, cmyc) and anti-apoptotic pathways (Bcl-XL, inhibitor of apoptosis proteins (IAPs). Additionally, by mediating the production of cyclooxygenase-2 (COX2), vascular endothelial growth factor (VEGF), interleukin (IL)8, intracellular cell adhesion molecule (ICAM), vascular cell adhesion molecule (VCAM) and matrix metalloproteinases (MMPs) it enhances angiogenesis and invasiveness [15]. In the cancer progression CDK4 kinase controls cell cycle transition from G1 to S phase [16]. The alteration in CDK4 function is demonstrated in colorectal cancer [17], breast cancer [18, 19], ovarian cancer [20, 21], lung cancer [22, 23], pancreatic cancer [24], and prostate cancer [25]. CDK4 knockout completely eliminates multiple endocrine neoplasia type 1-induced pituitary and pancreatic islet carcinogenesis [26, 27]. Among the players regulating inflammation and cell cycle, E2F1 and CyD1 are genes regulating the cyclin-dependent kinase (cdk) –retinoblastoma (–Rb) pathway [28]. Recent studies indicate that the molecular mechanisms employed by nuclear cdks support the expression of inflammatory mediators; some proinflammatory genes are overexpressed during the G1 phase of the cell cycle, and depend on cdks [29]. For example, the cytokine-induced recruitment of CDK6 to the nuclear chromatin fraction is associated with transcription factors of the NF-κB, STAT, and AP-1 families [16].

CyD1 is a proto oncogene and a primary regulator of cell proliferation, acting as a mitogen sensor that correlates cellular signaling mechanisms to the cell cycle. [30] Previous studies suggest that pRb, p16 and CyD1 are abnormally expressed in both colorectal adenomas and adenocarcinomas demonstrating a correlation between these factors. [31]. In inflammatory bowel diseases, colon-expressed CyD1 is aberrantly upregulated in both epithelial and immune cells [32]. Moreover, previous studies suggest that also E2F1 release has a role in the inflammatory response in patients with colitis, by the activation of P53 [33]. Also E2F1 is a key player in the inflammation control due to its relation to retinoblastoma protein (pRb). pRb controls cell cycle entry through the induction of transcription factors of the E2F family. After growth signals, pRb is hyper-phosphorylated by cdk complexes such as cyclin D/cdk4/6 and cyclin E/cdk2. [34] Overexpression of CyD1 is an early event in intestinal progression to cancer [35, 36] and dynamic expression of this cyclin is crucial for intestinal cell proliferation and maintenance of the transformed phenotype in mouse models and human colon cancer cells [37, 38]. However, in CyD1 knockout mice, intestinal tumors still developed, although in a reduced number [25]. This datum supports the hypothesis that CyD1 may not be essential for intestinal tumorigenesis, but it may act as a modifier gene [39] and it can be induced by NF-κB. This hyper-phosphorylation causes E2F release and transcription of other growth-associated genes. E2F1 seems to be a promising regulator in ovarian carcinoma and early studies suggest that E2F1 was significantly overexpressed in hepatocellular carcinoma and played a causative relationship with cell proliferation [40]. Also the cyclooxygenase 2 (COX2), a protein involved in inflammation, is increased in more aggressive forms of colorectal cancer, and is known to promote angiogenesis. [41] Accumulating evidence has also, demonstrated that cyclooxygenase-2 (COX-2) is involved in head and neck cancers, especially in nasopharyngeal carcinoma (NPC). [42]. COX-2 is a key isoenzyme in this biochemical cascade and is inducible by various oncogenic stimuli. A large volume of research data has shown that COX-2 is often upregulated in many malignant tumours, rendering it an attractive candidate target for cancer therapeutics [43]. One way to control the cell cycle are the siRNAs, powerful 20–25 bp double-stranded nucleotides, which can post-transcriptionally silence the gene expression modifying the related proteins expression levels [44]. siRNAs have a potential as drugs in the treatment of inflammation and cancer diseases but due to their hydrophilic nature and being negative charged they cannot cross cellular membrane and reach the nucleus [45]. Moreover, siRNAs are rapidly degraded by nucleases and cannot maintain their integrity for a long time [46]. Hence, there is a need for siRNA carrier systems to minimize siRNAs degradation and loss and, at the same time, increase their absorbance and bioavailability, favoring their biodistribution and penetration in the cellular compartment. Nanoliposomes-based drug delivery is an innovative and promising system to transport and control siRNAs release and overcome some of their limitations. [47–49]

In this regard, one of the main liposomes feature is their flexibility to link with site-specific ligands, molecules which allow achieving an active targeting, achieving the siRNA release only in the diseased tissue, without affecting the surrounding healthy tissue. Safety and efficacy are granted by smart-liposomes with tailored features regarding size, charge, load capability, and the ability to respond to stimuli. In fact, tailored carriers will take into account not only the drug features but also those of the tissue to be treated [50,51]. The intestinal epithelium is ideal for studying the mucosal homeostasis and cancer induction, because it is a rapidly proliferating tissue, and the mucosal homeostasis is dependent on the precise control of cellular proliferation within intestinal crypts. In particular, gut organ tissue explants are a suitable model to study the mechanisms that regulate the cell cycle progression and their potential control [52]. The aim of this study is to analyze the expression of CyD1 and E2F1 in colon tissues specimens from patients affected with Crohn’s disease after culture, and the efficacy of purposely formulated and produced siRNA vectors to deliver CyD1 and E2F1 siRNAs able to control inflammation progression.

## METHODOLOGY

### Tissue Culture

Blind to the characteristics of the patients, the in vitro organ cultures were performed by collecting intestinal specimen biopsy fragments from a healthy control and patients affected with Crohn’s disease during lower endoscopy. Healthy controls underwent colonoscopies because of colonic cancer screening, while Crohn’s disease patient underwent endoscopy as per routine follow-up. Exclusion criteria were age < 18 yrs, lack of informed consent, past or current therapy with immunosuppressant and/or biological agents, current therapy with steroids. A minimum of 14 fragments was needed for each experiment. The fragments were taken in the colon from uninflammed areas but next to the affected mucosa. Two fragments were oriented on Whatman paper and used for routine histology. The remaining 12 fragments were used for the experiment and placed in an ice-cold tissue-culture medium within 20 minutes and cultured as follows. To obtain the best results fragments are placed on a stainless-steel mesh in the well of an organ-culture dish containing culture medium with the epithelium facing upward. The dish is placed in a modular incubator chamber, supplemented with RPMI 1640 and 10% of fetal bovine serum, gassed with 5% CO2 and 95% O2, and then incubated at 37°C for a different time. [53] In this study we treated the colon biopsies with the siCy D1 and siE2F1 in presence or absence of EC-LPS (Lipopolysaccharides from Escherichia Coli, Sigma-Aldrich Co, MI; 2 μg/ml) and, after 24 h of incubation, we analyzed the tissue integrity and the CyD1 and E2F1 expression through Western Blotting technique. We also treated the colon biopsies with nanoliposomes conjugated with Rhodamine, incubating them for 4h. The study was approved from Campania Sud Ethical Committee.

### Immunohistochemical analysis

After ex vivo colon explant culture, serial 5 μm sections were stained with Hematoxylin and Eosin (H&E) and Masson’s Trichrome. Immunohistochemical (IHC) analysis if sections was perfomed incubating them for 40 minutes in methanol and 3% hydrogen peroxidase solution folled by a rinse in PBS. Samples were incubated 10 minutes in buffered citrate 0.01 M, pH 6, twice and rinsed in PBS. Sections were then treated with BSA (5%) for 10 minutes and finally incubated overnight with specific antibodies against CyD1 from NOVUS biologicals, used at 1:100 v/v and E2F1, Protein Tech, used at 1:50 v/v. Samples were then washed with PBS for 5 minutes and incubated with a labeled streptavidin biotin-peroxidase conjugate kit (Dako LSAB plus, cod. K0675, Dako Cytomation, Milan, Italy). After washing in PBS for 10 minutes, the sections were incubated with 3, 3-diaminobenzidine-tetrahydrochloride (DAB, Sigma Aldrich) for 1–3 minutes. The specificity of the immune reaction was revealed by the absence of the primary antibodies. Lastly, the samples were counterstained with Mayer’s Hematoxylin and observed under a Leica Microsystems.

### siRNA Selection

The sequences of two siRNA duplexes were synthesized by Invitrogen. The sense sequence for CyD1 was GCUAUUGGAGGAUCAGUUUTT, the sense sequence for E2F1 was GUCACGCUAUGAGACCUCATT. We also used Invitrogen negative (AMBIV NEG CTL cat. 4457287) and positive (AMBIV GAPDH SIRNA cat. 4457288) controls in our experiment to evaluate the efficacy of silencing.

### Nanoliposomes encapsulating siRNAs preparation and characterization

For nanoliposomes production, L-α-Phosphatidylcholine (PC) from egg yolk (CAS n. 8002-43-5), Cholesterol (CHO) (CAS n. 57-88-5), N-[1-(2,3-Dioleoyloxy)propyl]-N,N,N-trimethylammonium chloride (DOTAP) (CAS n. 132172-61-3) over 99% pure, Rhodamine B (CAS n. 81-88-9), potassium phosphate monobasic (CAS n.7778-77-0), sodium hydroxide (CAS n. 1310-73-2) and (hydroxymethyl)aminometane hydrochloride (Tris) were purchased from Sigma Aldrich (Milan, Italy) as dried powders and used without further purification. *Silencer*® Select siRNAs for CyD1 and E2F1 inhibition were purchased from Invitrogen; the sense sequences were the same as those above reported. All the other chemicals and reagents such as chloroform (CAS n. 67-66-3) and methanol (CAS n. 67-56-1) (Sigma Aldrich, Milan, Italy) used were of analytical grade. Nanoliposomes were prepared using thin film hydration method followed by duty cycle sonication as a size reduction method, developed by Barba and co-workers [50,51]. For the production of nanoliposomes encapsulating siRNAs sequences, DOTAP, CHOL and PC at 1.5:1:3 (mol: mol) ratio were chosen to achieve a positively charged lipid bilayer. Briefly, lipids were dissolved in 2 mL of chloroform/methanol 2:1 (vol/vol). Evaporation in a rotary evaporator removed the solvent (Heidolph, Laborota 4002 Control) and the produced lipid film was vacuum-dried for 3 hours at 50°C in a water bath. The dried lipid film was then hydrated at room temperature with 1 mL of Tris buffer solution (TBS - Tris(hydroxymethyl) aminometane hydrochloride 50 mM; sodium chloride 100 mM; pH 7.5), pre-warmed, containing 200 μl of 250 μM CyD1siRNA stock solution. The same procedure was done for E2F1siRNA encapsulation.

By this way, a sample of Multilamellar Vesicles (MLVs) encapsulating siRNAs at 50 μM final concentration was obtained using a DOTAP/siRNA charge ratio of 5:1 (+/−). The production of nanoliposomes was obtained by maintaining at room temperature for 2 hours the preparation containing MLVs and then sonicated it at 45% amplitude (treated volume: 1 mL) applying a duty cycle consisting of 2 irradiation rounds of 10 seconds, each followed by 20 seconds of pause to prevent thermal vesicle disruption. The nanoliposomes were stored at 4°C for one night, protected from light, and was then sonicated at 45% amplitude for four more rounds to achieve suitable small unilamellar vesicles SUVs. In this work the VCX 130 PB Ultrasonic Processors of Sonics & Materials Inc., USA instrument was used (its main features: maximum power 130 W, frequency 20 kHz; sonotrode tip length 137 mm; sonotrode tip diameter 3 mm) [54].

To morphologically characterize the produced nanoliposomes and study their uptake in tissue, a sample of empty nanoliposomes labeled with Rhodamine B was also prepared. The empty nanoliposomes were similarly produced, but the pocedure followed by hydratation of the thin lipid film with phosphate buffer solution (PBS) containing only Rhodamine B.

The lipids hydration solution contained potassium phosphate monobasic 0.2 M, sodium hydroxide 0.2 M and 5 μl of 5 mg/ml Rhodamine B stock solution to a pH 7.4 final solution.

Morphological characterization of empty SUVs was performed using optical microscopy (Axioplan 2-Image Zeiss, Jena, Germany) for fluorescent imaging. A 63× oil immersion objective was used, taking advantage of the fluorochrome Rhodamine B to label and visualize the vesicles.

The dimensional and zeta potential characterizations of SUVs containing siRNA sequences were done by Photon Correlation Spectroscopy (PCS). In particular, Dynamic Light Scattering analysis was performed by using the ZetasizerNano ZS (Malvern, UK) which incorporates noninvasive backscatter (NIBS) optics. The detection angle of 173 degrees was used. The resulting particle size distribution was plotted as the number of liposomes versus size. All the measurements were performed in triplicate. The results were expressed as average values.

### Nanoliposome-siRNAs complexes formation and stability

A retardation assay was performed to study the effective formation of the nanoliposomes-siRNAs complexes and their stability. A siRNAs silencer (1 μg) and nanoliposome-siRNA complexes, containing 1 μg of siRNAs were loaded into individual wells and subjected to electrophoresis on 1.5 % agarose gel (30 minutes at 80 V) in Tris/Borate/EDTA buffer (TBE) 0.5 M containing 45 mM Tris-borate and 1 mM EDTA. After staining with ethidium bromide (0.5 μg/ml), gels were viewed with UltraBright LED Transilluminator (Maestrogen, USA) at 470 nm.

### Nanoliposomes uptake in colon tissues

A fluorescence assay was used to study the nanoliposomes uptake in tissue. For this purpose 20 μl of nanoliposomes labeled with Rhodamine B were incubated in colon biopsy and then cultured for 4 hours; then biopsy tissues were frozen at − 80 °C. Subsequently, four-micrometer frozen tissue sections of biopsy samples were obtained and individually incubated for 5 minutes with DAPI (Invitrogen, diluted 1:1000 with deionized water) and then mounted with Mounting media (using glycerol and PBS at 5:1 v/v ratio). The liposome incorporation was studied by direct visualization of tissue section in fluorescence (Leica Microsystems).

### Ex vivo transfection in colon tissue with Invivofectamine® and nanoliposomes

Colon tissues were cultured as before described. We transfected the biopsies with CyD1siRNA and E2F1 siRNA at a final concentration of 5 nM using both the lipid mediated transfection carriers: Invivofectamine®® (commercial transfection reagent designed for systemic siRNA delivery) [55] and the produced nanoliposomes. As indicated by the manufacturer’s protocol for *Silencer*® Select siRNAs, a final concentration of 2 to 10 nM can be used to reduce mRNA levels for more than 80 % (Guidelines for transfection of mammalian cultured cells, Life Technologies).The amount of the commercial transfection agent was adjusted taking into account that for in vivo transfection in mice 10 μl/g of Invivofectamine® (Invivofectamine® 2.0, Invitrogen, Life Technologies, Cat. no. 1377501) are used and considering the recommended ratio of 2:1:1 (v/v) between Invivofectamine®, siRNA and the complexation buffer, included in the Invivofectamine® kit. Thus, for 4 μl of 50 μM siRNA stock solution, 4 μl of complexation buffer and 8 μl of Invivofectamine® were used. The siRNA was re-suspended with buffer complexation, and the solution was stirred vigorously before the addition of Invivofectamine®. The solution was stirred again and incubated at 50 ° C for 30 minutes before use. The nanoliposomes loaded with siRNA were used maintaining the same final concentration of 5 nM, thus for siRNA transfection, 4 μl of the nanoliposomes stock solution was used. Samples were treated as described in tissue culture, then frozen at −80 °C.

### Western blotting analysis

The colon specimen were lysed in buffer [50 mmol/L Tris-HCl (pH 8.0), 150 mmol/L NaCl, 0.1% SDS, 1% NP40] supplemented with protease and phosphatase inhibitors (CalbioChem). The proteins present in supernatants were quantified with the Breadford method assay (Biorad). Samples of 40 μg total proteins from colon preparation were applied to 10% sodium dodecyl sulfate/polyacrylamide gel electrophoresis (SDS-PAGE) and then transferred onto nitrocellulose membrane (Biorad). Blotting membranes were incubated with blocking solution with 5% non-fat dried milk powder dissolved in Tris-Buffered Saline Tween-20 (TBST) buffer (pH 7.5, 10 mmol / L Tris–HCl, 150 mmol /L NaCl, and 0.1% Tween20) for 1 h at room temperature, washed three times, and then incubated with rabbit monoclonal anti CyD1, (Novus Biologicals, cat. NB100-79920; 1:5000 dilution) in TBST overnight at 4 °C. After three washes with TBST buffer, we incubated the membranes for 1 hr with horseradish peroxidase–linked goat antirabbit secondary antibody (Immunoreagents Inc, 1:2000 dilution). The same procedure was done using rabbit monoclonal anti phosphoE2F1 (Proteintech, 1:50 dilution) and COX-2 (Cell Signaling 12282, 1:1000) Internal control was carried out using β-Actin rabbit antibody (ABM, cat. Y080025; 1:1000 dilution). The membranes were then processed by using Chemidoc (Bio-rad, Laboratories S.r.l., Segrate, MI) and treated with enhanced chemiluminescence western blotting detection reagents (Clarity Western ECL Substrate, Biorad, cat. 170-5060). The films were scanned, and the bands’ densitometry analysis was performed by using the *ImageJ* software for the relative level of the protein quantification.

### Cytotoxicity

We observed that the morphology and viability of tissues were maintained (PZ pathologist), that could suggest a non-toxicity of nanoliposomes and Invivofectamine® in tissues.

### Statistics

Values are expressed as mean together with SEM or SD as explicitly indicated.

## RESULTS

### Immunohistochemical analysis

**Fig. 1:**
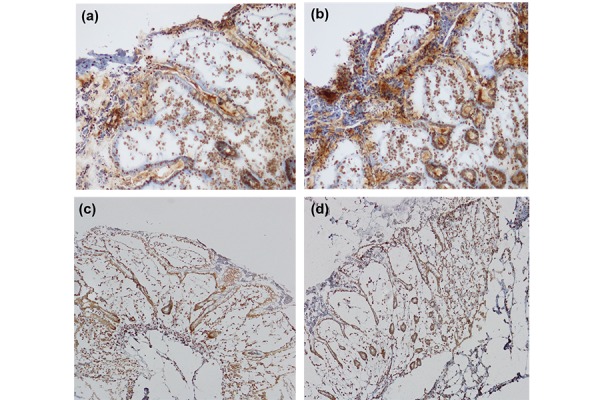
Microscope pictures of cultured colonic tissue after nuclear immunohistochemical analysis with the cyD1 antibody. (a) untreated tissue; (b) EC-LPS treated tissue. Microscope pictures of cultured colonic tissue after immunohistochemical analysis with the E2F1 antibody. (c) untreated tissue; (d) EC-LPS treated tissue. (Results from one representative experiment)

Cultures of human colon isolated from 6 different donors, two healthy controls (one male, one female) and four patients affected with Crohn’s disease (donor 1, white male, donor 2 white female; donor 3 white male, donor 4 white female) were used. A good integrity of tissues was observed through the immunohistochemical analysis. Both cyclins expressions increased after LPS treatment. Figures 1 show the CyD1 and E2F1 expression in a single patient as an example. The total number of Cyclin D1 and E2F1 positive cells is increased in all the patients after EC-LPS treatment (26% and 23%, respectfully in comparison with basal, untreated condition).

### Nanoliposomes characterization: morphology, size and zeta potential

**Fig.2:**
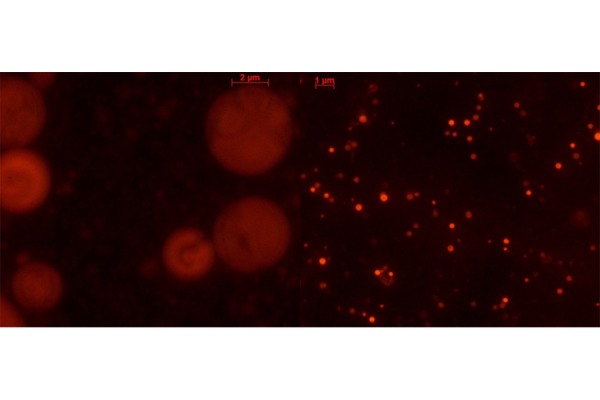
Fluorescence microscope pictures of liposomal vesicles labelled with Rhodamine B (Obj 63X). On the left, microliposomal vesicles before the sonication process; on the right, nanoliposomes after the duty cycle sonication size reduction process.

Spherical and defined liposomes were achieved through the production process before described. Figure 2 shows the nanoliposomal vesicles obtained before the sonication process and the nanoliposomal vesicles obtained after the duty cycle sonication process. The diameter size, polydispersity index (PDI) and zeta potential of cationic nanoliposomes encapsulating siRNAs were analyzed, and the obtained values are summarized in Table 1, presented as the average of three determinations. The nanometric structures produced have the right dimension useful for the Enhance Permeability and Retention Effect (EPR effect) which occurs in both inflammation and cancer conditions. The homemade lipid nanoparticles can diffuse and penetrate through tumor vascular fenestrations of 50 – 100 nm range size. Moreover, nanoliposomes are cationic as desired and as required to achieve an interaction with the negative siRNA molecule and also with the negatively charged plasma membrane.

### Nanoliposome-siRNAs complexes formation and stability

**Fig. 3:**
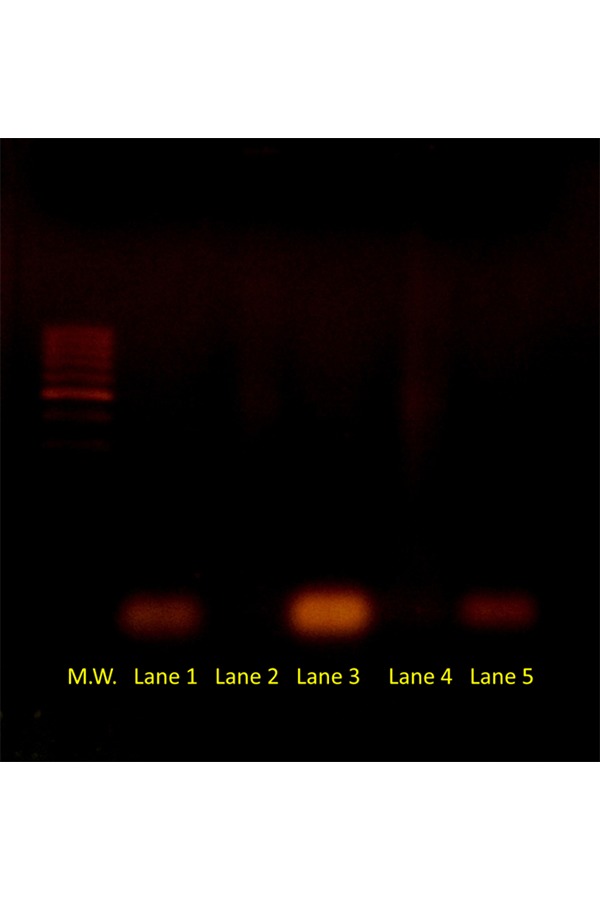
Band shift assay of siRNA binding interaction. In lane 1 siRNA CyD1 (1 μg) is in its naked form and the relative band is well visible; in lane 2 siRNA CyD1 (1 μg) is encapsulated in nanoliposomes and the band is not visible demonstrating the formation of a stable complex. In lane 3 siRNA E2F1 (1 μg) is in its naked form and the relative band is visible while in lane 4 siRNA E2F1 (1 μg) is encapsulated in nanoliposomes and the band is not visible demonstrating again the formation of a very stable complex. In the assay patients samples run in parallel with two controls: a 100 bp Ladder molecular weight visible on the left and a 21 bp ds-DNA molecule (simulating “Homo sapiens siRNA probe Luciferase”, 12833.4 g/mol) visible on the right.

As visible in figure 3, the retardation assay has shown the formation of very stable siRNA-SUVs complexes. In lane 2 and 4 any siRNA bands or bands smear are visible on an agarose gel, demonstrating the satisfactory encapsulation of siRNA molecules into nanoliposomes carriers projected including the positive charged DOTAP in the bilayer and using a 5/1 (+/−) charge ratio.

### Nanoliposomes uptake in colonic tissues

**Fig. 4:**
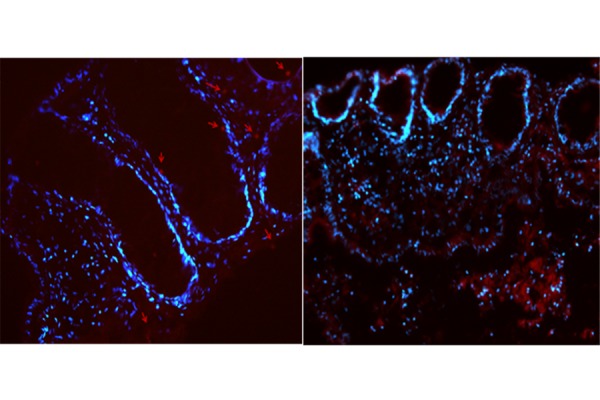
Fluorescence microscope pictures of Small Unilamellar Vesicles in cultured colonic tissue (Obj. 20X). Distribution of Rhodamine B-labeled SUVs in colonic mucosa sections in which nuclei are DAPI-labeled (time of incubation: 4 h) merge, Rhodamine and DAPI channels.

Fluorescence assay demonstrated the capacity of nanoliposomes to penetrate the colonic tissues. As shown in Figures 4, after four hours of incubation with Rhodamine labeled carriers, we observed the introduction of nanoliposomes in the biopsy tissue after the EC-LPS treatment. After four hours of incubation, nanoliposomes reached the basal membrane and lumen of crypts in the colonic epithelium.

### Effects of ex vivo transfection in colon tissue with Invivofectamine®® and nanoliposomes

**Fig. 5:**
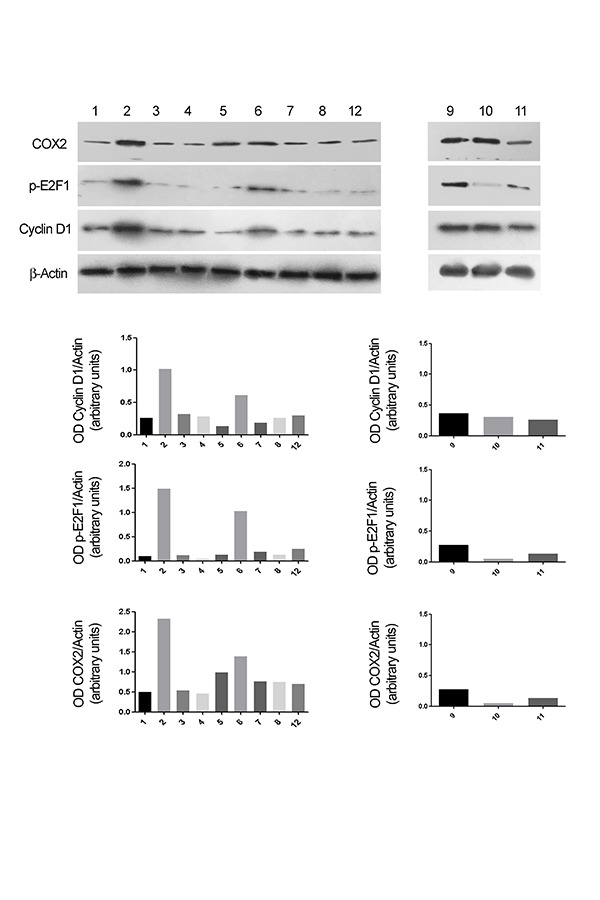
Evaluation of CyD1, E2F1 and COX-2 expression by Western Blot analysis of colon tissue samples from the patient 3 (PT3). Following the order of the analyzed samples: 1. Medium; 2. EC-LPS; 3. siRNA CyD1 transfected with Invivofectamine®; 4. siRNA CyD1 transfected in Nanoliposomes; 5. siRNA CyD1 in Invivofectamine® and EC-LPS; 6. siRNA CyD1 in Nanoliposomes and EC-LPS; 7. siRNA E2F1 transfected with Invivofectamine®; 8. siRNA E2F1 transfected in Nanoliposomes; 12. CTR – in Invivofectamine®; 9. siRNA E2F1 in Invivofectamine® and EC-LPS; 10. siRNA E2F1 in Nanoliposomes and EC-LPS; 11. CTR + in Invivofectamine®.

To study the changes of the protein levels during the inflammatory processes, we analyzed the effect of EC-LPS on CyD1, E2F1 and COX-2 expression in colon tissues. EC-LPS in the culture medium induced an increase of cyD 1, E2F1 and COX-2 expression in the mucosa. The silencing of cyD 1 and E2F1 showed a patient dependent response. Figure 5 shows an example of the response of E2F1 and cyD1 silencing both with invivofectamine and with nanoliposomes after EC-LPS treatment. COX-2 protein expression in the mucosa is reduced after CyD1 and E2F1silencing. (**Fig.5**).

## DISCUSSION

The present study investigated the potential utility of inhibiting E2F1 and CyD1 in modulating the inflammation process in human colonic tissue from patients affected with CD. These preliminary data demonstrated that gene silencing via RNA interference is possible and that tailored nanoliposomes may overcome the low penetration of siRNA across cell membranes which is a major obstacle for siRNA therapy.

The present study has, however, several limitations. First, this is a pilot study and the number of patients included is not adequate to draw definitive conclusions. Moreover, the included patients express differences in gender, age, duration and characteristics of the disease that may have influenced the results.

The silencing of CyD1 led to reduced protein expression in all patients. Silencing of E2F1 was differently effective in each patient. We demonstrated the inhibition of COX-2 expression after cyD1 and E2F1 silencing, substantiating other studies indicating a correlation between COX-2 and cyclins in cancer [56–58].

The present preliminary results make the basis for a potential gene therapy and “new smart nanovectors construction” able to treat IBD. Currently, commercial vectors can be used for all tissue indifferently. Thus, they lack tissue-specificity. If the aim is to reach specific cells and not others, there is the need for tissue-targeted systems. In the IBD therapy, both the siRNAs of choice and the nanocarriers should target specifically the inflamed colonic or ileal mucosa, reducing the systemic effect. Recently, our group identified siE2F1 nanoliposomes formulation (called siE2F1-SUV) with a better efficacy of uptake and silencing of E2F1 in cultured human biopsy of colonic mucosa and colon carcinoma cells [59]. However, in our opinion, the individual response is the key factor. In fact, the present data show that we obtained a patient-dependent response to the same silencing strategy. Ideally, in the future, researchers could achieve optimized nanoliposomes and siRNA specific for the single patient, to test for efficacy first on the patient’s biopsies.

## CONCLUSIONS

This study shows the preliminary results of the potential use of purposely formulated and produced siRNA vectors to deliver CyD1 and E2F1 siRNAs in control inflammation and cancer progression in IBD using the colon tissue culture in vitro model that represents a good example of translational medicine described as a tool to bring scientific knowledge “from bench to bedside” and then develop new therapies or medical procedures. Further studies are necessary to investigate their efficacy in the clinical practice.

## Figures and Tables

**Table 1:** Small Unilamellar Vesicles (SUVs) loaded with siCyD1, and siE2F1 mean diameter size, polydispersity index and zeta potential. Results are expressed as the average of three determinations; SD is the standard deviation.

Loaded sample	Diameter (nm) M ± SD	PDI M± SD	Zeta Pot. (mV) M± SD
siCyD1 SUVs	24.6 ± 5.0	0.42 ± 0.008	64.4 ± 1.85
siE2F1 SUVs	47.2 ± 4.9	0.33 ± 0.02	75.5 ± 0.45
